# Five‐Year CT‐Based Skeletal Muscle Decline Curves in Cirrhosis According to Liver Functional Reserve and Routinely Available Clinical Factors

**DOI:** 10.1002/jgh3.70472

**Published:** 2026-09-30

**Authors:** Suguru Miida, Hiroteru Kamimura, Hitoshi Yoshiji, Masahito Shimizu, Takumi Kawaguchi, Tetsuya Tsuji, Saori Endo, Hiroki Natsui, Hiroki Maruyama, Hiroyuki Abe, Tadashi Namisaki, Takao Miwa, Dan Nakano, Michiyuki Kawakami, Shuji Terai

**Affiliations:** ^1^ Division of Gastroenterology and Hepatology, Graduate School of Medical and Dental Sciences Niigata University Niigata Japan; ^2^ Department of Gastroenterology Nara Medical University Kashihara Japan; ^3^ Department of Gastroenterology/Internal Medicine Gifu University Graduate School of Medicine Gifu Japan; ^4^ Division of Gastroenterology, Department of Medicine Kurume University School of Medicine Fukuoka Japan; ^5^ Department of Rehabilitation Medicine Keio University School of Medicine Tokyo Japan

**Keywords:** longitudinal analysis, prediction model, sarcopenia, skeletal muscle loss, third lumbar vertebra skeletal muscle index

## Abstract

**Background:**

Sarcopenia is common in cirrhosis and is associated with poor outcomes. We aimed to identify factors associated with longitudinal skeletal muscle loss and to explore estimation of future L3 skeletal muscle index (L3‐SMI) using routinely available clinical variables.

**Methods:**

This retrospective longitudinal study included 175 patients with cirrhosis who underwent serial abdominal CT over five annual time points between 2010 and 2022. The primary analysis used a linear mixed‐effects model including annual time point, baseline Child–Pugh class, and their interaction. An exploratory linear regression model estimated Year 5 L3‐SMI.

**Results:**

In the primary model, L3‐SMI declined by −1.144 cm^2^/m^2^/year in baseline Child–Pugh class A. The time‐by‐class B interaction was not significant (*β* = 0.024; 95% CI, −0.250 to 0.298; *p* = 0.865), whereas class C was associated with an additional annual decline of −1.812 cm^2^/m^2^/year compared with class A (95% CI, −2.735 to −0.890; *p* = 0.0001). This association remained after adjustment for age, sex, baseline HCC status, and the time‐by‐HCC interaction. In the exploratory Year 5 model, baseline L3‐SMI was the dominant predictor; estimates for class C were interpreted cautiously because only five patients were baseline class C.

**Conclusions:**

Baseline hepatic functional reserve, particularly Child–Pugh class C, was associated with faster annual CT‐based L3‐SMI decline. The Year 5 prediction model is exploratory and requires external validation. Early sarcopenia assessment may facilitate risk stratification in patients with impaired hepatic reserve.

**Trial Registration:** UMIN Clinical Trials Registry: UMIN000051577.

## 1 Introduction

Sarcopenia is a progressive skeletal muscle disorder associated with frailty, falls, functional decline, impaired quality of life, and mortality [1, 2]. In cirrhosis, sarcopenia is common and prognostically important. A meta‐analysis of 22 cohort studies reported a prevalence of 37.5% and an independently increased mortality risk in patients with cirrhosis [3]. A recent CT‐based meta‐analysis of 39 studies confirmed a 44% prevalence and approximately twofold higher mortality risk, supporting the clinical relevance of EASL/AASLD CT‐based criteria [4].

These findings highlight the need to identify patients at high risk of progressive muscle loss. Liver rehabilitation, which includes exercise therapy, nutritional support, education, and psychosocial care, is increasingly recognized as a strategy to improve physical function, symptoms, prognosis, and quality of life in patients with steatotic liver disease, cirrhosis, and hepatocellular carcinoma (HCC) [5]. However, effective implementation requires longitudinal risk stratification.

The Japanese Society of Hepatology recommends grip strength, bioelectrical impedance analysis, and CT‐derived skeletal muscle area at the third lumbar vertebra normalized by height squared as the L3 skeletal muscle index (L3‐SMI) for sarcopenia assessment [6]. The EASL guidelines also recognize cross‐sectional imaging as an objective method for sarcopenia assessment in cirrhosis [7]. The EASL/AASLD CT cutoffs were proposed by Carey et al. [8], and their prognostic value has been supported by recent meta‐analysis [4].

Despite the usefulness of CT‐based L3‐SMI, repeated quantification may not be feasible in all clinical settings, and determinants of individual skeletal muscle trajectories remain incompletely defined. Cirrhosis‐related sarcopenia is influenced by disease‐specific mechanisms such as hyperammonaemia, altered autophagy, impaired protein metabolism, and reduced anabolic signaling [9]. We therefore aimed to identify factors associated with progressive skeletal muscle decline in cirrhosis and to develop a clinically usable framework for estimating longitudinal L3‐SMI using routine clinical information and CT‐based muscle assessment.

## 2 Patients and Methods

### 2.1 Study Design and Population

This longitudinal retrospective study included patients with cirrhosis followed at Niigata University Medical and Dental Hospital between January 2010 and December 2022. Cirrhosis was diagnosed by board‐certified hepatologists based on clinical, laboratory, and imaging findings. Patients were eligible if they had continuous follow‐up for at least 5 years and at least one abdominopelvic CT examination annually during the 5‐year observation period. A total of 175 patients met these criteria.

### 2.2 Clinical Care and Cohort Characteristics

CT was performed primarily for HCC surveillance or post‐treatment monitoring, and laboratory tests and physical examinations were performed approximately concurrently with CT. Hepatic functional reserve was assessed using Child–Pugh class, modified albumin‐bilirubin (mALBI) grade, and Model for End‐Stage Liver Disease (MELD) score. Some patients had a history of locoregional therapy, systemic chemotherapy, or surgery for HCC, but all had an Eastern Cooperative Oncology Group performance status of 0. Alcohol intake was defined as regular consumption exceeding 20 g/day of ethanol for women and 30 g/day for men.

Because serial CT was required, this cohort should be interpreted as a clinically relevant subgroup of patients with cirrhosis undergoing HCC surveillance or controlled post‐treatment follow‐up, rather than as an unselected cirrhosis population. Although completion of 5‐year follow‐up and preserved performance status reduced the likelihood of uncontrolled cancer‐related cachexia, the influence of previous HCC, recurrence, or anti‐cancer treatment on skeletal muscle trajectories could not be fully excluded.

### 2.3 Skeletal Muscle Mass Assessment

Skeletal muscle area at L3 was measured on axial CT images using SliceOmatic software (version 5.0, TomoVision Inc., Montreal, Canada). Muscles were quantified within −29 to +150 HU [10], with manual correction when necessary. Three physicians blinded to clinical information independently reviewed the images, and the final L3 muscle area was determined by consensus.

### 2.4 Parameter Definitions and Longitudinal Assessment

The L3‐SMI derived from the first CT was defined as baseline L3‐SMI or L3‐SMI (1y), and Child–Pugh class at that time was recorded as the baseline Child–Pugh grade. CT was performed at approximately 1, 2, 3, 4, and 5 years from the first annual CT assessment. Because CT intervals varied, L3‐SMI values were standardized to yearly time points by linear interpolation between consecutive CT examinations, as previously reported [11]. The primary longitudinal outcome was annualized change in L3‐SMI across the five annual CT time points. The worst Child–Pugh grade and worst mALBI grade were used only for exploratory descriptive analyses, not as baseline predictors. The annualization procedure and analytical hierarchy are shown in Figure 1.

**FIGURE 1:**
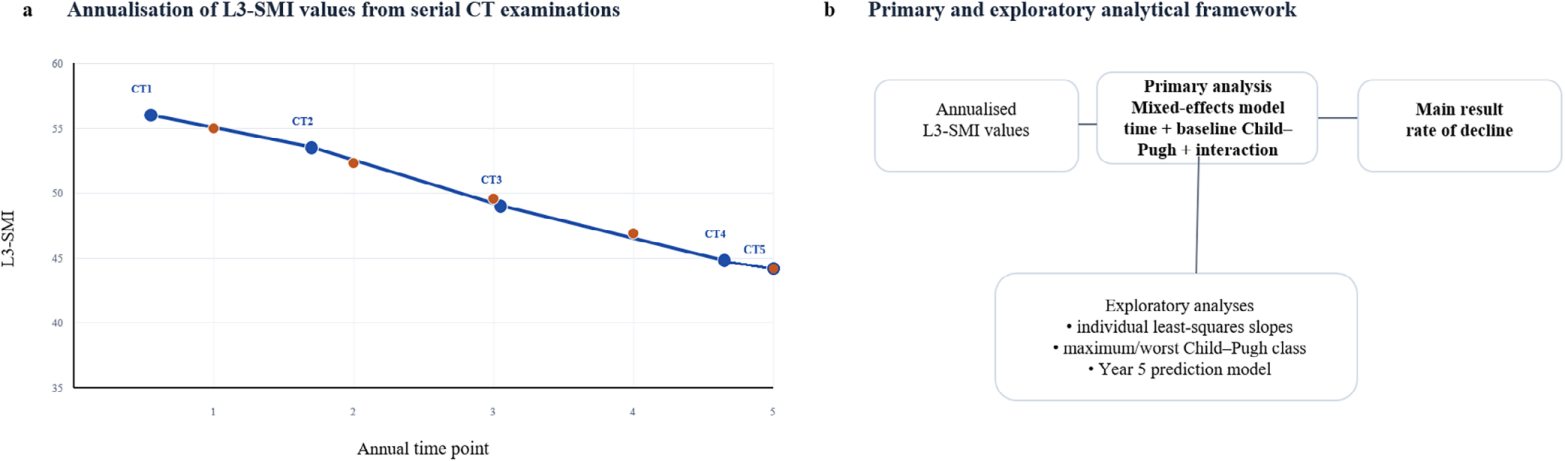
Annualization of CT‐based L3‐SMI measurements and longitudinal analysis framework. (a) Blue circles indicate observed L3‐SMI values at the actual CT examination dates, whereas orange circles indicate annualized L3‐SMI values estimated at prespecified yearly time points by linear interpolation between consecutive CT measurements. The blue line represents linear interpolation between observed CT measurements. When an annualized time point coincided with an observed CT examination, the symbols overlap. (b) The analytical hierarchy is shown. The primary analysis used a mixed‐effects model including annual time point, baseline Child–Pugh class, and the time‐by‐Child–Pugh interaction. Individual least‐squares slopes, maximum/worst Child–Pugh class during follow‐up, and the Year 5 prediction model were treated as exploratory analyses. CT, computed tomography; L3‐SMI, skeletal muscle index at the third lumbar vertebral level.

### 2.5 MELD Score

The MELD score was calculated using total bilirubin, creatinine, and the international normalized ratio as follows: MELD = 3.78 × ln(bilirubin [mg/dL]) + 11.2 × ln(INR) + 9.57 × ln(creatinine [mg/dL]) + 6.43. Values < 1.0 were set to 1.0, and creatinine was capped at 4.0 mg/dL, including dialysis. MELD is commonly used to assess liver disease severity and transplant priority [12]. MELD scores were categorized into quartiles: Q1 < 9.485, Q2 9.485 to < 12.171, Q3 12.171 to < 15.178, and Q4 ≥ 15.178.

### 2.6 Physical Activity Assessment

Physical activity (PA) was assessed in 57 patients using the short International Physical Activity Questionnaire [13]. Weekly energy expenditure was calculated as MET‐min/week using 8.0, 4.0, and 3.3 MET for vigorous activity, moderate activity, and walking, respectively [14]. Participants were classified as low (< 600 MET‐min/week), moderate (600–3000 MET‐min/week), or high (> 3000 MET‐min/week). Hepatic functional deterioration was defined as progression from Child–Pugh A to B/C or from Child–Pugh B to C during follow‐up.

### 2.7 Statistical Analysis

Linear mixed‐effects models were fitted with L3‐SMI as the dependent variable and a patient‐specific random intercept to account for repeated measurements within individuals. To directly evaluate differences in longitudinal skeletal muscle decline, the primary model included annual time point, baseline Child–Pugh class, and the time‐by‐Child–Pugh interaction as fixed effects. Additional models were adjusted for age and sex, and a sensitivity model further included baseline HCC status and the time‐by‐HCC interaction. Alternative longitudinal models used baseline mALBI grade or MELD quartiles instead of Child–Pugh class to assess whether the association between liver functional reserve and annual L3‐SMI decline was consistent across different liver reserve indices. Sex was coded as female = 0 and male = 1; Child–Pugh class A, mALBI Grade 1, and MELD Q1 were used as the respective reference categories.

Individual least‐squares slopes of L3‐SMI were calculated for each patient and summarized according to the maximum Child–Pugh class observed during follow‐up; because this classification was determined after baseline, these analyses were treated as exploratory and descriptive and were not used for causal inference. An exploratory linear regression model was used to estimate Year 5 L3‐SMI using baseline L3‐SMI, sex, BMI, age, and baseline Child–Pugh class. The original prediction equation derived in the initial analysis was retained for continuity, but the model was considered hypothesis‐generating and not used for primary inference. Variables were selected a priori based on clinical availability, biological plausibility, and avoidance of overfitting.

Comparisons presented in Table 2 were descriptive and exploratory; *p*‐values were not adjusted for multiplicity and should therefore be interpreted cautiously. Linear mixed‐effects models were fitted using R version 4.4.1, and other analyses were performed using GraphPad Prism version 9.0. A two‐sided *p*‐value < 0.05 was considered statistically significant.

In addition, exploratory cross‐sectional linear models were used to examine associations between baseline SMI and age or liver reserve indices, with adjustment for sex, albumin, prothrombin time, creatinine, loop diuretic use, and l‐carnitine supplementation. These adjusted analyses were reported in Table S1 and were kept separate from the primary longitudinal inference. Unadjusted cross‐sectional age–SMI associations according to liver reserve index are shown in Figure S1.

### 2.8 Ethical Considerations

This study was conducted in accordance with the Declaration of Helsinki and approved by the Institutional Review Board of Niigata University (approval number: 2023‐0009). The requirement for consent for retrospective data use was waived through an opt‐out approach, and written informed consent was obtained from patients who completed the IPAQ.

## 3 Results

### 3.1 Patient Background and L3‐SMI According to Liver Functional Reserve

Table 1 summarizes the baseline characteristics of the 175 patients. The most common etiology was hepatitis C virus infection, followed by alcohol‐related liver disease, metabolic dysfunction‐associated steatohepatitis, and hepatitis B virus infection. Table 2 presents baseline characteristics according to baseline Child–Pugh class. These comparisons were descriptive and unadjusted for multiplicity.

**TABLE 1:** Baseline characteristics of the study population overall baseline characteristics.

	*N* (%)
Age (years), median (range)	69 (39–86)
Sex (M), *n*	106 (60.6)
Etiology	*n* (%)
Hepatitis C	57 (32.6)
Alcohol	46 (26.3)
MASH	28 (16.0)
Hepatitis B	23 (13.1)
PBC	10 (5.7)
AIH	4 (2.3)
Others	7 (4.0)
Baseline Child–Pugh class (A/B/C)	128 (73.1)/42 (24.0)/5 (2.9)
Maximum Child–Pugh class during follow‐up (A/B/C)	77 (44.0)/62 (35.4)/36 (20.6)
Baseline mALBI Grade (1/2a/2b/3)	57 (32.6)/39 (22.3)/66 (37.7)/13 (7.4)
Maximum mALBI grade during follow‐up (1/2a/2b/3)	17 (9.7)/26 (14.9)/71 (40.6)/61 (34.9)

Abbreviations: AIH, autoimmune hepatitis; MASH, metabolic dysfunction‐associated steatohepatitis; mALBI, modified albumin–bilirubin grade; PBC, primary biliary cholangitis.

**TABLE 2:** Baseline characteristics stratified by baseline Child–Pugh class.

	Factors (mean ± SD or *n* (%)) (*n* = 175)			*p*		
	Child A (*n* = 128)	Child B (*n* = 42)	Child C (*n* = 5)	A versus B	B versus C	A versus C
BMI (kg/m^2^)	24.5 ± 5.1	24.2 ± 4.3	27.7 ± 11.2	0.945	0.330	0.367
Age (years)	69 ± 10	67 ± 10	61 ± 12	0.350	0.486	0.196
Sex (male)	75 (58.6%)	28 (66.7%)	3 (60.0%)	0.370	> 0.999	> 0.999
HCC‐positive, *n* (%) Stage (I/II/III/IV), *n*	91 (71.1%) 11/30/30/20	26 (61.9%) 4/5/8/9	1 (20.0%) 0/0/0/1	0.342	0.148	0.034
Hb (g/dL)	13.0 ± 1.8	11.4 ± 2.5	11.3 ± 1.0	< 0.001	0.991	0.148
PLT (× 10^4^/μL)	12.9 ± 5.4	9.6 ± 5.8	8.1 ± 4.9	0.004	0.846	0.163
TP (g/dL)	7.5 ± 0.6	7.0 ± 0.8	6.7 ± 1.1	< 0.001	0.695	0.060
Alb (g/dL)	3.9 ± 0.4	3.1 ± 0.4	2.3 ± 0.8	< 0.001	0.001	< 0.001
AST (U/L)	51 ± 66	65 ± 43	71 ± 40	0.367	0.980	0.747
ALT (U/L)	41 ± 60	39 ± 26	57 ± 59	0.965	0.750	0.792
LDH	211 ± 80	255 ± 79	373 ± 78	0.006	0.005	< 0.001
ALP (IFCC)	106 ± 45	133 ± 70	152 ± 56	0.009	0.741	0.132
PT (%)	89.1 ± 14.6	61.3 ± 15.3	40.8 ± 3.6	< 0.001	0.010	< 0.001
T‐Bil (mg/dL)	0.9 ± 0.4	1.8 ± 0.9	5.1 ± 3.2	< 0.001	< 0.001	< 0.001
D‐Bil (mg/dL)	0.1 ± 0.1	0.4 ± 0.3	2.0 ± 1.5	< 0.001	< 0.001	< 0.001
γ‐GTP (U/L)	104 ± 193	142 ± 235	67 ± 43	0.550	0.716	0.915
mALBI score	−2.53 ± 0.38	−1.69 ± 0.32	−0.77 ± 0.86	< 0.001	< 0.001	< 0.001
MELD score	10.33 ± 4.02	15.37 ± 3.74	20.94 ± 2.38	< 0.001	0.009	< 0.001
FIB‐4 index	4.85 ± 2.69	10.13 ± 7.58	10.65 ± 7.59	< 0.001	0.969	0.016
HbA1c (%)	6.4 ± 4.2	5.7 ± 1.2	5.0 ± 0.4	0.537	0.917	0.712
Na (mEq/L)	139.4 ± 2.5	138.1 ± 3.0	138.0 ± 3.4	0.019	0.997	0.486
K (mEq/L)	4.1 ± 0.5	4.0 ± 0.4	4.1 ± 0.5	0.055	0.791	0.971
ChE (U/L)	227 ± 72	143 ± 52	101 ± 44	< 0.001	0.380	< 0.001
BUN (mg/dL)	16.2 ± 8.0	15.9 ± 6.6	17.2 ± 13.5	0.973	0.936	0.960
Cre (mg/dL)	0.83 ± 0.56	0.83 ± 0.28	0.76 ± 0.20	> 0.999	0.961	0.953
TG (mg/dL)	112 ± 60	92 ± 40	85 ± 20	0.123	0.956	0.524
LDL‐C (mg/dL)	94.4 ± 27.1	74.8 ± 30.8	55.8 ± 24.6	< 0.001	0.331	0.008
HDL‐C (mg/dL)	56.2 ± 19.3	46.2 ± 16.1	24.3 ± 16.9	0.013	0.003	0.067
NH_3_ (μg/dL)	58.8 ± 28.2	67.4 ± 30.6	86.4 ± 19.4	0.257	0.346	0.092
L3‐SMI (male) (cm^2^/m^2^)	48.2 ± 5.3	46.1 ± 7.6	40.8 ± 3.7	0.317	0.308	0.087
L3‐SMI (female) (cm^2^/m^2^)	39.3 ± 7.4	35.2 ± 4.7	38.3 ± 1.0	0.180	0.832	0.976
BCAA, *n* (%)	42 (32.8%)	25 (61.0%)	5 (100.0%)	0.002	0.147	0.005
Lactulose‐based laxatives, *n* (%)	9 (7.0%)	6 (14.6%)	3 (60.0%)	0.202	0.044	0.005
Loop diuretics, *n* (%)	19 (14.8%)	15 (36.6%)	2 (40.0%)	0.006	1.000	0.177
MRAs, *n* (%)	21 (16.4%)	17 (41.5%)	4 (80.0%)	0.002	0.163	0.004
Tolvaptan, *n* (%)	2 (1.6%)	2 (4.9%)	2 (40.0%)	0.248	0.053	0.007
l‐carnitine supplementation, *n* (%)	5 (3.9%)	4 (9.8%)	4 (80.0%)	0.223	0.002	< 0.001
Rifaximin, *n* (%)	3 (2.3%)	6 (14.6%)	2 (40.0%)	0.007	0.203	0.011
Antihypertensive agents, *n* (%)	60 (46.9%)	14 (34.1%)	0 (0.0%)	0.205	0.303	0.064
Dyslipidaemia, *n* (%)	15 (11.7%)	3 (7.3%)	0 (0.0%)	0.567	> 0.999	> 0.999
Antidiabetic agents, *n* (%)	33 (25.8%)	5 (12.2%)	0 (0.0%)	0.086	> 0.999	0.332
Corticosteroids, *n* (%)	6 (4.7%)	0 (0.0%)	1 (20.0%)	0.338	0.109	0.240
UDCA, *n* (%)	50 (39.1%)	19 (46.3%)	3 (60.0%)	0.467	0.659	0.387
Alcohol intake, *n* (%)	23 (18.0%)	11 (26.8%)	1 (20.0%)	0.263	> 0.999	> 0.999

Abbreviations: ALP, alkaline phosphatase; ALT, alanine aminotransferase; AST, aspartate aminotransferase; BCAA, branched‐chain amino acids; BMI, body mass index; Bil, bilirubin; BUN, blood urea nitrogen; ChE, cholinesterase; Cre, creatinine; FIB‐4, fibrosis 4; Hb, hemoglobin; HbA1c, hemoglobin A1c; HCC, hepatocellular carcinoma; HDL, high‐density lipoprotein; LDL, low‐density lipoprotein; L3‐SMI, skeletal muscle index at the L3 vertebral level; LDH, lactate dehydrogenase; MELD, model for end‐stage liver disease; MRAs, mineralocorticoid receptor antagonists; PLT, platelet count; PT, prothrombin time; TP, total protein; UDCA, ursodeoxycholic acid.

Only five patients had baseline Child–Pugh class C. As Child–Pugh class progressed, albumin, cholinesterase, and prothrombin time decreased, whereas total bilirubin, ALBI score, MELD score, and FIB‐4 index increased. Significant intergroup differences were also observed for hemoglobin, platelet count, LDH, ALP, LDL‐cholesterol, and HDL‐cholesterol, whereas AST, ALT, and sex‐specific L3‐SMI did not differ significantly; sodium was slightly lower in class B than in class A in this unadjusted descriptive comparison. Exploratory unadjusted cross‐sectional associations between age and L3‐SMI according to Child–Pugh class, mALBI grade, and MELD quartiles are shown in Figure S1. In exploratory adjusted cross‐sectional models, older age was inversely associated with baseline SMI (*β* approximately −0.512 to −0.529 across the Child–Pugh, mALBI, and MELD models), whereas male sex and albumin were positively associated with baseline SMI. Loop diuretic use was consistently associated with lower baseline SMI (*β* approximately −1.233 to −1.286). These adjusted models are summarized in Table S1.

### 3.2 Primary Longitudinal Mixed‐Effects Analysis of Annual L3‐SMI Decline

The primary mixed‐effects model included annual time point, baseline Child–Pugh class, and their interaction terms (Panel A of Table 3). In baseline Child–Pugh class A, L3‐SMI declined by −1.144 cm^2^/m^2^/year (95% CI, −1.276 to −1.011; *p* < 0.0001). The time‐by‐Child–Pugh class B interaction was not significant (*β* = 0.024; 95% CI, −0.250 to 0.298; *p* = 0.865), whereas the time‐by‐Child–Pugh class C interaction was significant (*β* = −1.812; 95% CI, −2.735 to −0.890; *p* = 0.0001). The estimated annual declines were approximately −1.144, −1.120, and −2.956 cm^2^/m^2^/year for baseline Child–Pugh classes A, B, and C, respectively. These standardized slope‐based trajectories are illustrated in Figure 2a.

**TABLE 3:** Primary mixed‐effects models evaluating annual L3‐SMI decline according to baseline liver functional reserve.

Variable	Estimated coefficient	95% confidence interval	*p*
*Panel A: Primary model including baseline Child–Pugh class and time interaction*			
Intercept	45.167	43.710 to 46.624	< 0.0001
Annual time point	−1.144	−1.276 to −1.011	< 0.0001
Child–Pugh class B	1.941	−0.993 to 4.875	0.195
Child–Pugh class C	6.142	−1.450 to 13.734	0.113
Time × Child–Pugh class B	0.024	−0.250 to 0.298	0.865
Time × Child–Pugh class C	−1.812	−2.735 to −0.890	0.0001
*Panel B: Secondary model including baseline mALBI grade and time interaction* ^a^			
Intercept	51.510	43.793 to 59.227	< 0.0001
Annual time point	−1.177	−1.379 to −0.976	< 0.0001
mALBI Grade 2a	0.215	−2.718 to 3.148	0.886
mALBI Grade 2b	2.594	0.011 to 5.177	0.049
mALBI Grade 3	2.309	−2.052 to 6.669	0.299
Time × mALBI Grade 2a	0.020	−0.300 to 0.341	0.901
Time × mALBI Grade 2b	0.078	−0.201 to 0.357	0.582
Time × mALBI Grade 3	−0.402	−0.926 to 0.123	0.133
Age	−0.174	−0.279 to −0.069	0.001
Sex (male)	8.104	5.992 to 10.216	< 0.0001
*Panel C: Secondary model including MELD quartiles and time interaction* ^b^			
Intercept	52.606	45.019 to 60.193	< 0.0001
Annual time point	−1.146	−1.348 to −0.943	< 0.0001
MELD Q2	−0.000	−2.947 to 2.947	1.000
MELD Q3	1.012	−2.106 to 4.131	0.525
MELD Q4	2.934	−0.349 to 6.217	0.080
Time × MELD Q2	0.194	−0.115 to 0.504	0.219
Time ×MELD Q3	−0.259	−0.583 to 0.065	0.118
Time × MELD Q4	−0.068	−0.408 to 0.272	0.694
Age	−0.177	−0.283 to −0.071	0.001
Sex (male)	7.281	4.957 to 9.606	< 0.0001

Abbreviations: mALBI, modified albumin‐bilirubin grade; MELD, model for end‐stage liver disease.

^a^ Models in Panels B and C were adjusted for age and sex. Reference categories were mALBI Grade 1 and MELD Q1.

^b^ The primary model used baseline Child–Pugh class because it was prespecified as the most clinically interpretable hepatic reserve variable.

**FIGURE 2:**
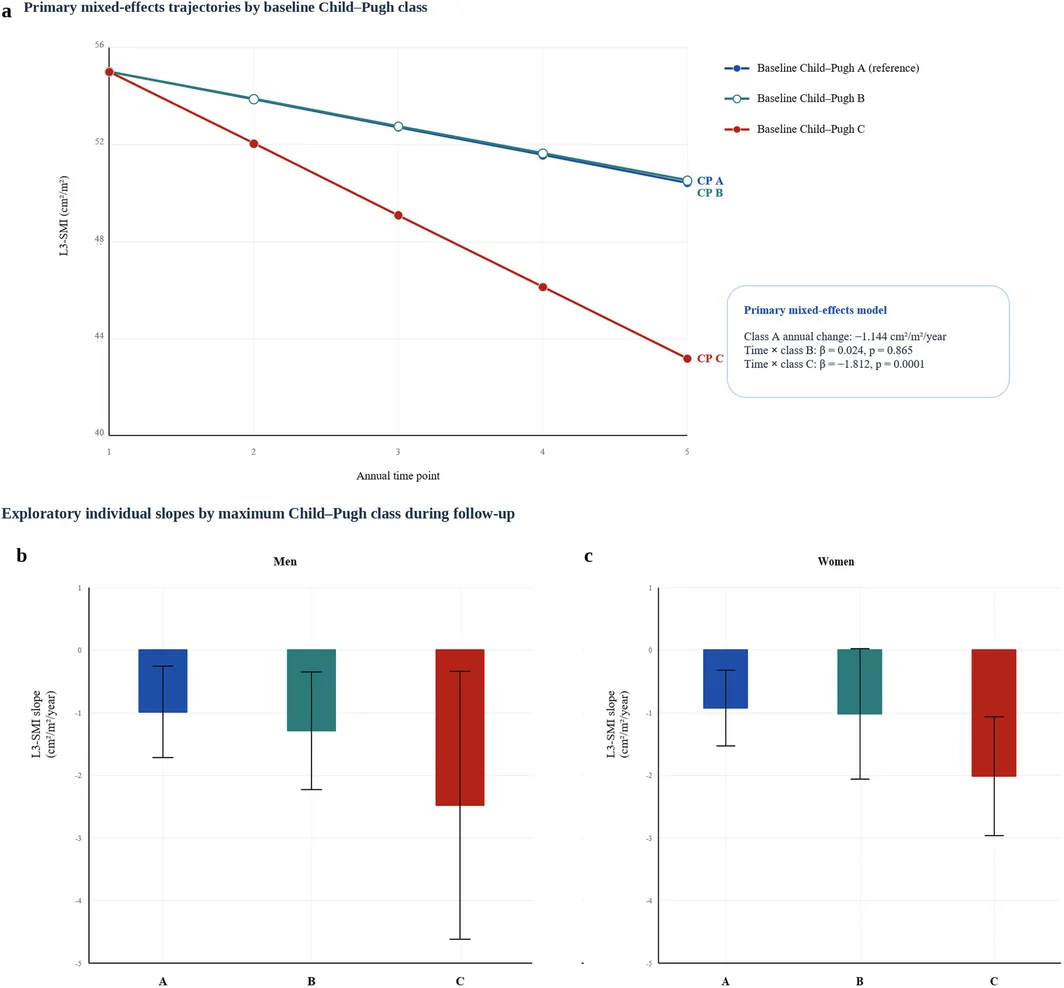
Primary longitudinal trajectories and exploratory individual slopes according to Child–Pugh class. (a) Standardized trajectories illustrate the annual L3‐SMI decline rates estimated from the primary mixed‐effects model according to baseline Child–Pugh class. All trajectories were standardized to a common Year 1 L3‐SMI value to visualize differences in decline rate rather than baseline absolute values. Child–Pugh class A declined by −1.144 cm^2^/m^2^/year. The time‐by‐Child–Pugh class B interaction was not significant (*β* = 0.024; *p* = 0.865), indicating that the class A and B slopes were nearly identical, whereas the time‐by‐Child–Pugh class C interaction was significant (*β* = −1.812; *p* = 0.0001), indicating faster annual L3‐SMI decline in baseline class C. (b, c) Exploratory individual least‐squares slopes of L3‐SMI are summarized according to the maximum/worst Child–Pugh class observed during follow‐up for men and women, respectively. Because maximum/worst Child–Pugh class was determined after baseline, panels b and c are descriptive and should not be interpreted causally. The maximum Child–Pugh groups comprised 77 class A, 62 class B, and 36 class C patients. L3‐SMI, skeletal muscle index at the third lumbar vertebral level.

The direction of the findings was unchanged after adjustment for age and sex (Panel A of Table 4). In a sensitivity model including baseline HCC status and the time‐by‐HCC interaction, the time‐by‐Child–Pugh class C interaction remained significant (*β* = −1.963; 95% CI, −2.901 to −1.026; *p* < 0.0001), whereas the time‐by‐HCC interaction did not reach significance (*β* = −0.225; 95% CI, −0.476 to 0.027; *p* = 0.080) (Panel B of Table 4). Models using baseline mALBI grade or MELD quartiles showed overall annual decline, but their time interaction terms were less consistent than those for baseline Child–Pugh class (Panels B and C of Table 3). Sensitivity analyses are summarized in Figure 3a,b.

**TABLE 4:** Sensitivity analyses for the longitudinal mixed‐effects and exploratory Year 5 prediction models.

Panel A: Model adjusted for age and sex			
Variable	Estimated coefficient	95% confidence interval	*p*
Intercept	53.458	45.811 to 61.105	< 0.0001
Annual time point	−1.145	−1.280 to −1.010	< 0.0001
Child–Pugh class B	0.894	−1.658 to 3.446	0.492
Child–Pugh class C	4.661	−1.952 to 11.274	0.167
Time × Child–Pugh class B	0.030	−0.247 to 0.307	0.832
Time × Child–Pugh class C	−1.828	−2.756 to −0.900	0.0001
Age	−0.187	−0.293 to −0.081	0.001
Sex (male)	7.782	5.650 to 9.914	< 0.0001

Panel B: Model adjusted for age, sex, baseline HCC status, and time‐by‐HCC interaction			
Variable	Estimated coefficient	95% confidence interval	*p*
Intercept	52.840	45.205 to 60.475	< 0.0001
Annual time point	−0.989	−1.210 to −0.769	< 0.0001
Child–Pugh class B	0.806	−1.742 to 3.354	0.535
Child–Pugh class C	4.381	−2.294 to 11.055	0.198
Baseline HCC status	−0.847	−3.305 to 1.611	0.499
Time × Child–Pugh class B	0.022	−0.254 to 0.298	0.877
Time × Child–Pugh class C	−1.963	−2.901 to −1.026	< 0.0001
Time × HCC status	−0.225	−0.476 to 0.027	0.080
Age	−0.173	−0.281 to −0.064	0.002
Sex (male)	8.161	5.952 to 10.371	< 0.0001

Panel C: Exploratory sensitivity analyses of the Year 5 prediction model			
Sensitivity analysis	Coefficient	95% confidence interval	*p*
Child–Pugh B/C combined	0.389	−1.283 to 2.061	0.645
Child–Pugh C excluded: class B	0.710	−0.959 to 2.380	0.401
HCC‐adjusted prediction model: HCC status	−0.641	−2.242 to 0.960	0.429

*Note:* Sex was coded as female = 0 and male = 1; Child–Pugh class A and HCC‐negative status were reference categories. Panel C summarizes sensitivity analyses for the exploratory Year 5 prediction model.

Abbreviation: HCC, hepatocellular carcinoma.

**FIGURE 3:**
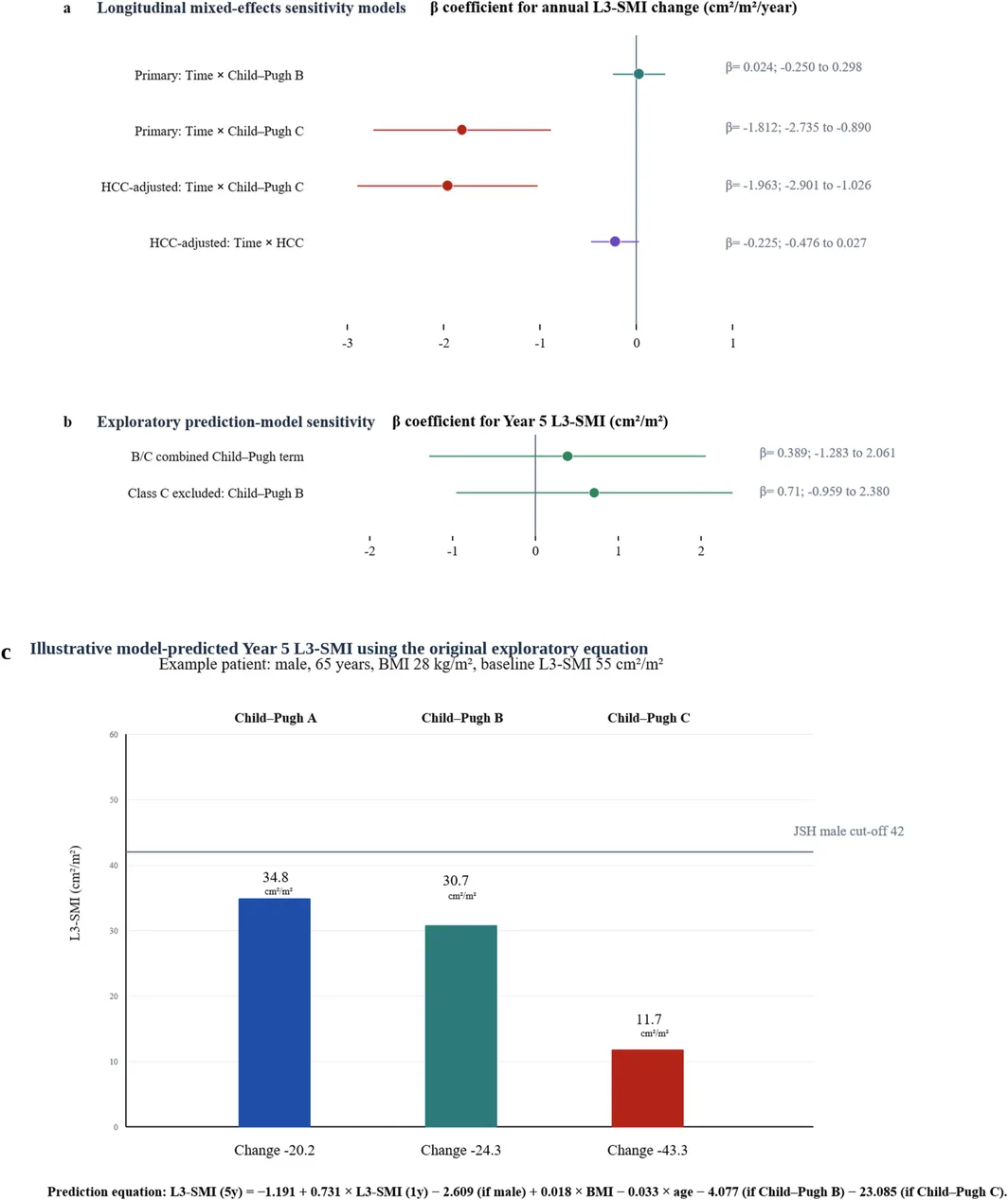
Sensitivity analyses and exploratory Year 5 prediction model. (a) Forest plots show time‐by‐covariate interaction coefficients from the longitudinal mixed‐effects models. Negative coefficients indicate a faster annual decline in L3‐SMI relative to the reference group. The time‐by‐Child–Pugh class C interaction remained significant after adjustment for baseline HCC status, whereas the time‐by‐HCC interaction was not statistically significant. (b) Sensitivity analyses of the exploratory Year 5 prediction model combining Child–Pugh classes B/C or excluding class C. Neither sensitivity estimate was statistically significant. These sensitivity models were separately re‐fitted and do not replace the coefficients of the original exploratory Year 5 prediction equation. Points represent regression coefficients and horizontal lines represent 95% confidence intervals; the vertical line indicates *β* = 0. (c) Model‐predicted L3‐SMI at Year 5 is shown for an example male patient aged 65 years with BMI 28 kg/m^2^ and baseline L3‐SMI 55 cm^2^/m^2^ using the original exploratory prediction equation. Predicted Year 5 L3‐SMI values were 34.8, 30.7, and 11.7 cm^2^/m^2^ for Child–Pugh classes A, B, and C, respectively. Because only five patients had baseline Child–Pugh class C, the class C estimate should be interpreted cautiously and not used for primary inference. The gray horizontal line indicates the Japan Society of Hepatology CT‐based male L3‐SMI cutoff of 42 cm^2^/m^2^. BMI, body mass index; HCC, hepatocellular carcinoma; JSH, Japan Society of Hepatology; L3‐SMI, skeletal muscle index at the third lumbar vertebral level.

### 3.3 Exploratory Comparison of Individual Slopes Using the Maximum Child–Pugh Class Observed During Follow‐Up

In an exploratory descriptive analysis, individual L3‐SMI slopes were estimated by least‐squares regression and summarized according to the maximum Child–Pugh class observed during follow‐up. Because this classification was determined during the observation period, it was not used as a baseline predictor and should not be interpreted causally. Using this descriptive classification, the cohort included 77 Child–Pugh A, 62 Child–Pugh B, and 36 Child–Pugh C patients. Among men, annual L3‐SMI decline was −0.991 ± 0.727, −1.296 ± 0.940, and −2.485 ± 2.140 cm^2^/m^2^/year in Child–Pugh A, B, and C, respectively. Among women, corresponding values were −0.928 ± 0.606, −1.021 ± 1.042, and −2.017 ± 0.951 (Figure 2b,c).

### 3.4 Alcohol Etiology and Physical Activity

Among men who remained Child–Pugh A, muscle decline was numerically greater in alcohol‐related cirrhosis than in other aetiologies, but the difference was not significant (−1.09 ± 0.82 vs. −0.94 ± 0.69 cm^2^/m^2^/year; *p* = 0.51) (Figure S2a). PA data were available for 57 patients: 30 low, 17 moderate, and 10 high. Hepatic functional deterioration occurred in 22.2% (6/27) of the moderate/high PA group and 40.0% (12/30) of the low PA group (*p* = 0.149) (Figure S2b).

### 3.5 Exploratory Model for Estimating Year 5 L3‐SMI

Table S2 shows the exploratory linear regression model for estimating Year 5 L3‐SMI. Baseline L3‐SMI was significantly associated with Year 5 L3‐SMI (*β* = 0.731; 95% CI, 0.317–1.145; *p* = 0.0006). Compared with Child–Pugh class A, baseline Child–Pugh class C was associated with lower predicted Year 5 L3‐SMI (*β* = −23.085; 95% CI, −40.821 to −5.349; *p* = 0.0111), whereas class B was not significant (*β* = −4.077; 95% CI, −11.022 to 2.868; *p* = 0.2480). Sex, BMI, and age were retained as clinically relevant covariates but were not individually significant. Because only five patients had baseline Child–Pugh class C, estimates involving class C in the prediction model were interpreted cautiously and not used for primary inference.

The regression equation was retained from the original exploratory model: Predicted L3‐SMI (5y) = −1.191 + 0.731 × L3‐SMI (1y)—2.609 (if male) + 0.018 × BMI—0.033 × age—4.077 (if Child–Pugh B)—23.085 (if Child–Pugh C). Apparent model performance in the derivation cohort showed *R* ^2^ 0.92, adjusted *R* ^2^ 0.91, and RMSE 6.46 cm^2^/m^2^. Sensitivity analyses showed that the Child–Pugh term was not significant when classes B and C were combined (*β* = 0.389; *p* = 0.645) or when class C was excluded (Child–Pugh B: *β* = 0.710; *p* = 0.401). Therefore, the equation is presented as an exploratory risk‐stratification framework rather than as a validated prediction model. An illustrative example based on this exploratory equation is shown in Figure 3c.

## 4 Discussion

To our knowledge, this is the first longitudinal study to evaluate long‐term rates of skeletal muscle decline using CT in patients with cirrhosis with a primary mixed‐effects framework that directly tested annual L3‐SMI decline according to baseline liver functional reserve.

The present cohort should be interpreted as a real‐world cirrhosis cohort under HCC surveillance and/or controlled post‐treatment follow‐up rather than as a fully unselected cirrhosis population. Sensitivity analysis adjusted for baseline HCC status and the time‐by‐HCC interaction, and the association between baseline Child–Pugh class C and faster annual L3‐SMI decline remained. However, detailed time‐dependent information on HCC recurrence and anti‐cancer treatment was not uniformly available; residual confounding by HCC‐related factors cannot be fully excluded.

Skeletal muscle mass physiologically declines by approximately 1.4% annually in the general older population [15]. By contrast, more rapid skeletal muscle loss has been reported in patients with cirrhosis [11]. This accelerated decline is likely attributable to disease‐specific mechanisms, including protein‐energy malnutrition caused by reduced dietary intake, malabsorption, impaired hepatic protein synthesis, and metabolic dysregulation [16, 17, 18, 19]. In addition, with the increasing prevalence of obesity worldwide, sarcopenic obesity has become a highly relevant phenotype in chronic liver disease [20]. In the present cohort, the mean BMI was ≥ 24 kg/m^2^ in Child–Pugh A and B and ≥ 27 kg/m^2^ in Child–Pugh C, suggesting that obesity may coexist with skeletal muscle depletion in patients with cirrhosis [21].

A consistent finding across the longitudinal models was that L3‐SMI declined over time and that older age was associated with lower L3‐SMI. In the primary Child–Pugh interaction model, baseline Child–Pugh class C showed significantly faster annual decline than class A, whereas class B did not. This supports the clinical importance of both aging and advanced hepatic dysfunction in skeletal muscle depletion among patients with cirrhosis.

The association between hepatic functional reserve and L3‐SMI decline depended on the scoring system. Baseline Child–Pugh class showed the clearest interaction with time, whereas the interaction terms for mALBI grade and MELD quartiles were less consistent. The Child–Pugh classification may capture clinically relevant features such as ascites and encephalopathy in addition to laboratory parameters, whereas mALBI and MELD may reflect partly different dimensions of liver function [22].

The sex‐specific findings warrant careful interpretation. In the baseline stratified analysis, sex‐specific L3‐SMI did not differ significantly across Child–Pugh classes, although numerical differences were observed. In the exploratory sex‐specific slope analysis, men with maximum Child–Pugh class C showed numerically steeper annual declines in L3‐SMI than those with class A or B, whereas the pattern was less clear among women. These exploratory findings should be interpreted cautiously and do not establish a sex‐specific interaction. Nevertheless, the observed numerical pattern suggests that the relationship between hepatic dysfunction and muscle loss may differ by sex and warrants evaluation in larger cohorts. Men with cirrhosis may be particularly vulnerable to rapid muscle depletion despite greater absolute muscle mass, whereas women may have lower baseline muscle reserve and distinct hormonal influences on muscle maintenance [23, 24]. Future studies should consider sex‐specific patterns when evaluating sarcopenia progression in cirrhosis.

Albumin was consistently positively associated with baseline SMI in the exploratory adjusted cross‐sectional models. This finding is consistent with the close relationship between hepatic synthetic function, nutritional status, and preservation of skeletal muscle mass. Albumin contributes to oncotic pressure, amino acid transport, and protein turnover, and hypoalbuminemia may reflect or contribute to a catabolic state that promotes muscle loss in cirrhosis [16, 17, 18, 19]. Accordingly, lower albumin may be closely linked to skeletal muscle depletion, supporting nutritional assessment and optimization of protein–energy balance in cirrhosis.

Loop diuretic use was also consistently associated with lower baseline SMI in the exploratory adjusted cross‐sectional models. This association is biologically plausible and consistent with previous reports suggesting that loop diuretics may contribute to muscle loss through electrolyte depletion, mitochondrial dysfunction, or increased urinary nitrogen excretion [25, 26, 27]. However, because we assessed only the presence or absence of loop diuretic use and did not evaluate dose or duration, causality cannot be inferred. These findings support careful evaluation of diuretic therapy in patients at risk of sarcopenia and future studies examining dose‐dependent effects and alternative agents such as mineralocorticoid receptor antagonists or tolvaptan.

In the subgroup analysis of men who remained Child–Pugh A during follow‐up, alcohol‐related cirrhosis was associated with numerically greater muscle loss, but the difference was not statistically significant. Because this subgroup was small, the analysis should be interpreted cautiously. Nonetheless, the direction of the trend is biologically plausible, given the known roles of ethanol‐related mitochondrial dysfunction and endotoxin‐associated pathways in alcohol‐related muscle wasting [28, 29]. Our findings do not establish alcohol etiology as an independent determinant of muscle decline, but they suggest that this relationship warrants further investigation in larger cohorts with more detailed longitudinal assessment of drinking behavior.

Physical activity may also have an important protective role. In the IPAQ subgroup, hepatic functional deterioration was numerically more frequent in the low‐activity group than in the moderate/high‐activity group, although the difference did not reach significance. This negative result should be interpreted in the context of limited sample size. The observed trend is compatible with evidence that physical activity in cirrhosis is generally lower than in healthy controls [30] and supports further prospective evaluation of structured exercise interventions.

With the increasing prevalence of MASH‐related cirrhosis [31], exercise therapy is receiving growing attention. In fatty liver disease, exercise can improve steatosis and metabolic dysfunction, and current guidelines support weight reduction and lifestyle intervention in overweight or obese patients [32, 33, 34, 35, 36, 37]. In addition, experimental and epidemiologic studies suggest that regular physical activity may contribute to a lower risk of HCC and liver‐related mortality [38, 39].

At the same time, caution remains necessary in patients with advanced cirrhosis because exercise may increase portal pressure or aggravate renal dysfunction in susceptible individuals [40, 41]. Nevertheless, prior interventional studies suggest that carefully selected and appropriately supervised exercise programs can be implemented safely in patients with compensated cirrhosis [42, 43]. In this context, a clinically useful model for identifying patients at high risk of skeletal muscle loss could help guide the timing and intensity of individualized exercise‐based interventions.

Our exploratory prediction framework suggests that estimation of future L3‐SMI from baseline clinical status may help identify patients who require individualized intervention. We retained the original exploratory prediction equation to preserve continuity with the derivation analysis, but the revised interpretation clearly positions it as preliminary and hypothesis‐generating. Baseline L3‐SMI was the strongest predictor of predicted 5‐year L3‐SMI, and baseline Child–Pugh class C was associated with a markedly lower predicted value; however, only five patients were baseline Child–Pugh class C. In addition, sensitivity analyses combining classes B and C or excluding class C did not show a significant Child–Pugh effect in the prediction model. Accordingly, the equation should not be regarded as a validated clinical prediction tool, and external validation is required before broader clinical implementation.

This study has some limitations. First, this was a single‐center retrospective study of patients who underwent serial CT mainly for HCC surveillance or posttreatment monitoring. Although baseline HCC status was included in sensitivity analysis, detailed time‐dependent data on HCC recurrence, anti‐cancer treatment type, timing, and cumulative treatment burden were not uniformly available. Second, only five patients had baseline Child–Pugh class C; estimates involving baseline class C should therefore be interpreted cautiously, particularly in the exploratory Year 5 prediction model. Third, disease duration was not incorporated into the analyses. Fourth, the physical activity and alcohol subgroup analyses were limited by sample size. Finally, multiple variables were compared descriptively in Table 2 without adjustment for multiplicity. Future prospective multicenter studies with larger numbers of patients with advanced cirrhosis, detailed HCC treatment trajectories, and external validation are needed. In addition, the adjusted cross‐sectional models for baseline SMI, including albumin and loop diuretic use, were exploratory and should not be interpreted as evidence of longitudinal causality.

## 5 Conclusions

In this study, baseline Child–Pugh class C was associated with faster annual decline in CT‐based L3‐SMI in patients with cirrhosis, and this association remained after adjustment for baseline HCC status. The Year 5 prediction model was exploratory and mainly driven by baseline L3‐SMI, underscoring the importance of early sarcopenia assessment. These findings provide a clinically usable framework for risk stratification and support CT‐based muscle assessment to identify patients with impaired hepatic reserve who may require more intensive monitoring and individualized management.

## Funding

This study was partially supported by the Japan Agency for Medical Research and Development (AMED) under Grant No. 24fk0210128h0002.

## Conflicts of Interest

Hitoshi Yoshiji received honoraria from Otsuka Pharmaceutical Co. Ltd., Asuka Pharmaceutical Co. Ltd., Gilead Sciences, Novel Pharmaceutical Co., Taisho Pharmaceutical Co. Ltd., AbbVie Inc., and Eisai Co. Ltd., and received research funding from Asuka Pharmaceutical Co. Ltd. and AbbVie Inc. Takumi Kawaguchi received honoraria from ASKA Pharmaceutical Co. Ltd., Taisho Pharmaceutical Co. Ltd., Kowa Company Ltd., AbbVie GK, Eisai Co. Ltd., EA Pharma Co. Ltd., Nippon Boehringer Ingelheim Co. Ltd., Sumitomo Pharma Co. Ltd., Novo Nordisk Pharma Ltd., Otsuka Pharmaceutical Co. Ltd., and Janssen Pharmaceutical K.K. The other authors declare no conflicts of interest.

## Supporting information


**Table S1:** Exploratory adjusted cross‐sectional models for baseline SMI.
**Table S2:** Exploratory linear regression model for estimating Year 5 L3‐SMI.

**Figure S1:** Unadjusted cross‐sectional age–SMI relationships by liver reserve index, Panels (a), (b), and (c) show Child–Pugh class, mALBI grade, and MELD quartiles, respectively. Scatter plots show the unadjusted cross‐sectional relationship between age and SMI, with descriptive regression lines for each liver reserve category. These analyses were based on 175 patients and are exploratory. Adjusted cross‐sectional models are reported separately in Table S1 and were not used for primary longitudinal inference. mALBI, modified albumin–bilirubin; MELD, Model for End‐stage Liver Disease; SMI, skeletal muscle index.

**Figure S2:** Exploratory analyses of alcohol‐related muscle decline and physical activity, (a) Individual annual L3‐SMI decline was compared between alcohol‐related cirrhosis and other aetiologies among men who remained Child–Pugh class A during follow‐up. (b) The proportion of patients with hepatic functional deterioration was compared between the low physical activity group and the moderate/high physical activity group. These analyses are exploratory and descriptive, and *p*‐values were not adjusted for multiplicity. L3‐SMI, skeletal muscle index at the third lumbar vertebral level; PA, physical activity.

## Data Availability

The data that support the findings of this study are available from the corresponding author upon reasonable request, subject to approval by the Institutional Review Board of Niigata University. The data are not publicly available due to privacy and ethical restrictions.
