# Supplementary material for: Five‐Year CT‐Based Skeletal Muscle Decline Curves in Cirrhosis According to Liver Functional Reserve and Routinely Available Clinical Factors
Source: JGH Open. 2026 Sep 30;10(10):e70472. doi: 10.1002/jgh3.70472 (PMC13624706; doi:10.1002/jgh3.70472)
Supplement: Supplementary file 1 [file JGH3-10-e70472-s001.docx]

**Supplementary Table S1. Exploratory adjusted cross-sectional models for baseline SMI**

Models examined baseline SMI using age and each of the three prespecified liver reserve indices used in the manuscript framework, with adjustment for sex, albumin, prothrombin time, creatinine, loop diuretic use, and L-carnitine supplementation. These analyses were exploratory and based on 175 patients at Niigata University.

**Panel A. Model including age, Child–Pugh class, and adjustment covariates**

| **Variable** | **Estimated coefficient** | **95% confidence interval** | **p-value** |
| --- | --- | --- | --- |
| Intercept | 72.647 | 65.830 to 79.465 | <0.0001 |
| Age | -0.524 | -0.608 to -0.439 | <0.0001 |
| Child–Pugh class B | -0.091 | -0.789 to 0.607 | 0.799 |
| Child–Pugh class C | -1.596 | -2.801 to -0.391 | 0.010 |
| Sex (male) | 6.829 | 4.494 to 9.163 | <0.0001 |
| Albumin | 1.179 | 0.648 to 1.710 | <0.0001 |
| Prothrombin time | -0.015 | -0.033 to 0.003 | 0.106 |
| Creatinine | -0.026 | -0.515 to 0.463 | 0.918 |
| Loop diuretic use (yes) | -1.275 | -2.106 to -0.445 | 0.003 |
| L-carnitine supplementation (yes) | -1.075 | -2.262 to 0.111 | 0.076 |

**Panel B. Model including age, mALBI grade, and adjustment covariates**

| **Variable** | **Estimated coefficient** | **95% confidence interval** | **p-value** |
| --- | --- | --- | --- |
| Intercept | 72.368 | 65.050 to 79.685 | <0.0001 |
| Age | -0.529 | -0.614 to -0.443 | <0.0001 |
| mALBI grade 2a | -0.204 | -0.917 to 0.508 | 0.574 |
| mALBI grade 2b | -0.067 | -1.028 to 0.893 | 0.891 |
| mALBI grade 3 | -0.600 | -2.184 to 0.983 | 0.458 |
| Sex (male) | 6.823 | 4.477 to 9.168 | <0.0001 |
| Albumin | 1.271 | 0.454 to 2.088 | 0.002 |
| Prothrombin time | -0.010 | -0.027 to 0.006 | 0.220 |
| Creatinine | -0.057 | -0.550 to 0.436 | 0.820 |
| Loop diuretic use (yes) | -1.286 | -2.126 to -0.445 | 0.003 |
| L-carnitine supplementation (yes) | -1.102 | -2.300 to 0.096 | 0.072 |

**Panel C. Model including age, MELD quartiles, and adjustment covariates**

| **Variable** | **Estimated coefficient** | **95% confidence interval** | **p-value** |
| --- | --- | --- | --- |
| Intercept | 71.529 | 64.829 to 78.228 | <0.0001 |
| Age | -0.512 | -0.598 to -0.425 | <0.0001 |
| MELD Q2 | -0.488 | -1.140 to 0.165 | 0.144 |
| MELD Q3 | -0.562 | -1.390 to 0.267 | 0.185 |
| MELD Q4 | -1.180 | -2.205 to -0.154 | 0.025 |
| Sex (male) | 7.081 | 4.736 to 9.426 | <0.0001 |
| Albumin | 1.394 | 0.936 to 1.852 | <0.0001 |
| Prothrombin time | -0.018 | -0.036 to 0.001 | 0.058 |
| Creatinine | 0.012 | -0.483 to 0.507 | 0.963 |
| Loop diuretic use (yes) | -1.233 | -2.069 to -0.398 | 0.004 |
| L-carnitine supplementation (yes) | -1.126 | -2.316 to 0.064 | 0.064 |

Abbreviations: mALBI, modified albumin–bilirubin; MELD, Model for End-stage Liver Disease; SMI, skeletal muscle index.

**Supplementary Table S2. Exploratory linear regression model for estimating Year 5 L3-SMI**

| Variable | Estimated coefficient | 95% confidence interval | p-value |
| --- | --- | --- | --- |
| Intercept | -1.191 | -31.977 to 29.594 | 0.9392 |
| Baseline L3-SMI (1y) | 0.731 | 0.317 to 1.145 | 0.0006 |
| Sex (male) | -2.609 | -9.667 to 4.449 | 0.4665 |
| BMI | 0.018 | -0.075 to 0.111 | 0.7034 |
| Age | -0.033 | -0.349 to 0.283 | 0.8368 |
| Child-Pugh class B | -4.077 | -11.022 to 2.868 | 0.2480 |
| Child-Pugh class C | -23.085 | -40.821 to -5.349 | 0.0111 |

BMI, body mass index; L3-SMI, skeletal muscle index at the third lumbar vertebral level. Female sex and Child-Pugh class A were used as reference categories. This exploratory equation was retained from the original derivation analysis. Because only five patients had baseline Child-Pugh class C, estimates involving class C are unstable and should be interpreted cautiously.
